# Supplementary material for: RamanBot: Versatile high throughput Raman system
Source: PLoS One. 2026 Jan 13;21(1):e0334679. doi: 10.1371/journal.pone.0334679 (PMC12798987; doi:10.1371/journal.pone.0334679)
Supplement: S1 File — This document includes the detailed RamanBot scanning workflow and coordinate registration methods (Section A), an assessment of optical crosstalk in clear microplates (Section B), a comparison of the Cartesian motion architecture versus traditional motorized stages (Section C), and a kinematic analysis of motion delays and margin time optimization (Section D). (PDF) [file pone.0334679.s001.pdf]

Detailed system characterization and experimental workflows

Section A: Detailed workflow

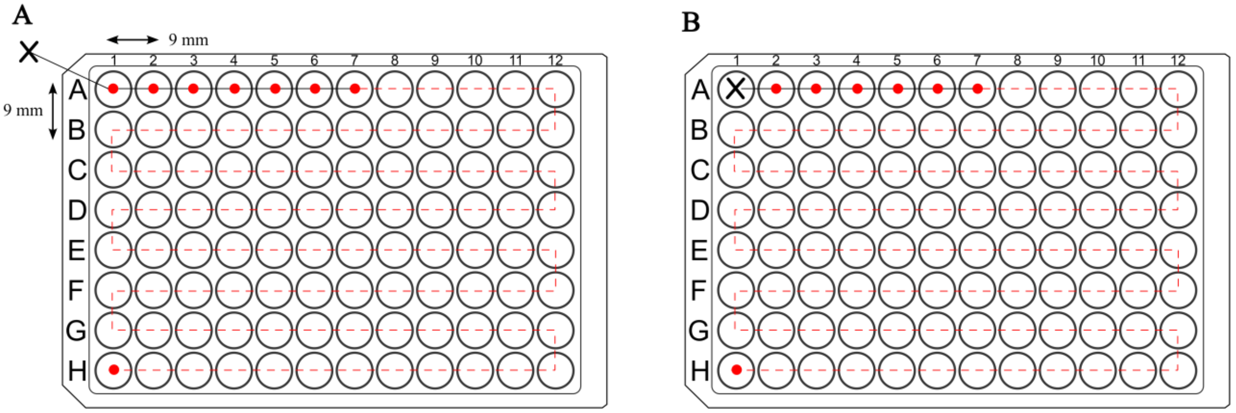

Figure A1: Schematic of RamanBot scanning trajectories across a 96-well microplate. (A) Absolute positioning mode, where the path initiates from a fixed machine origin ('X') external to the plate. (B) Relative positioning mode, where the first sample (well A1, marked by a circled 'X') serves as the starting reference. Red dots indicate discrete spectral acquisition points along the serpentine scanning path (dashed line).

Sample geometry is mapped manually using one of two coordinate registration modes, as illustrated in Figure A1. The workflow is defined as follows:

1. **Coordinate Registration:** The sample container is secured to the print bed, and the starting coordinates are registered:
    a. Absolute Positioning (Figure A1A): The (X, Y, Z) location of the first sample (e.g., well A1) is determined relative to the RamanBot's fixed hardware origin (0,0,0) (indicated by the 'X' outside the microplate). All subsequent sample positions are defined by their absolute machine coordinates.
    b. Relative Positioning (Figure A1B): The RamanBot objective is manually focused on the center of the first sample (indicated by the circled 'X' in well A1). This position is established as the local reference point for the current run. Subsequent movements to adjacent wells are defined by relative offsets (e.g., the 9 mm well pitch shown in Figure A1).
2. Timing Parameterization: The total pause time per sample is calculated as the sum of the acquisition time, and a stability margin.

3. Instruction Generation: Based on the registered coordinate list and calculated timing, a custom script generates a G-code file. This file commands the stepper motors to traverse the defined sample array (e.g., the serpentine path shown in Figure A1 and pause for acquisition (red dots)).
4. Synchronization: Simultaneously, an acquisition schedule is generated for the spectrometer. This schedule synchronizes the spectral acquisition triggers to align strictly with the mechanical pause windows defined in the G-code.
5. Execution and Data Retrieval: The G-code and acquisition schedules are executed in parallel. Upon completion, the spectral data is aggregated and spatially mapped to the corresponding sample coordinates for analysis.

Section B: Clear microplate crosstalk

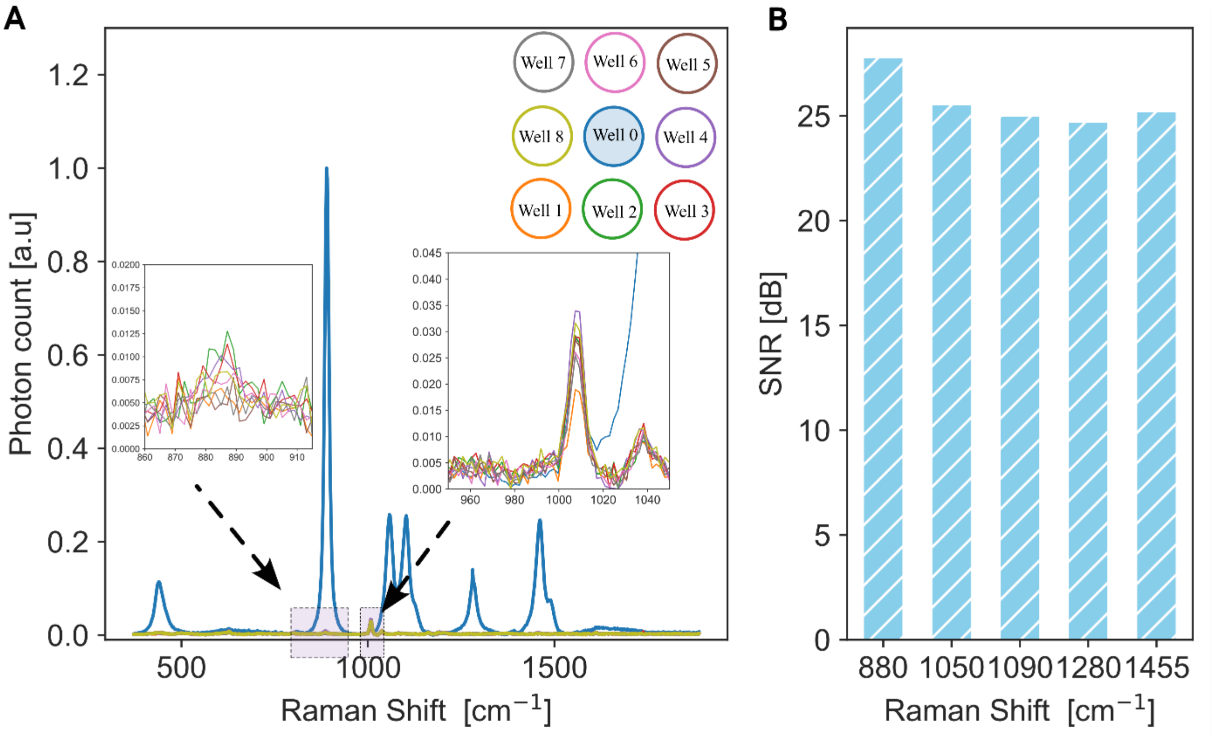

Figure B1: Clear microplate cross-talk evaluation with ethanol sample. A: Nine Raman spectra collected from a 3×3 arrangement of microplate wells, where the central well (well 0) contains ethanol and the surrounding wells are filled with water. The left inset highlights the spectral region near 880 cm−1, revealing the ethanol-associated peak and demonstrating detectable ethanol signal into adjacent wells. The right inset highlights the spectral region around 1000 cm-1, revealing the effect of the microplate spectrum on the collected spectra. B: The signal-to-noise ratio calculated at the major ethanol peaks (880, 1050, 1090, 1280, and 1455 cm−1) for the sample in the central well.

In this experiment, Raman spectra were acquired from a 3×3 grid in a clear microplate, where the central well (Well 0) contained ethanol (330 ul) and the surrounding wells (Wells 1–8) contained water (330 ul). Figure B1A displays the intense ethanol spectrum observed in Well 0, contrasted with the significantly attenuated signals from the surrounding wells. The left inset highlights the spectral region near the dominant ethanol peak at 880 cm^{-1}. A faint trace of this peak is visible in the water-filled wells, which can be attributed to two factors: first, laser-induced heating may increase ethanol volatility, leading to vapor cross-contamination in adjacent wells; second, the optical transparency of the microplate

may permit scattered light to waveguide through the substrate and be collected by the objective lens. Additionally, the right inset of Figure B1 reveals a prominent polystyrene peak around 1000 cm^{-1}. If not adequately subtracted, this background signal could confound quantitative analysis by overlapping with nearby reference peaks. Figure B1B shows the signal to noise ratio (SNR) computed for ethanol dominant peaks (880, 1050, 1090, 1280, and 1455 cm−1). The SNR levels reported are comparable to the black microplate SNR levels.

Section C: Cartesian Motion System vs. Traditional Motorized Stage

While traditional motorized microscopy stages excel in sub-micron repeatability and structural rigidity, they are often constrained by limited travel ranges and high costs. In contrast, the Cartesian motion system—adapted from additive manufacturing architectures—offers distinct operational advantages for high-throughput screening. Primarily, it provides a significantly expanded working envelope (250x250x210 mm) at a fraction of the cost of comparable microscopy stages. This architecture prioritizes macroscopic speed and volumetric range over sub-micrometer precision, a trade-off aligned with the requirements of rapid, large-area screening. Furthermore, the extensive open-source ecosystem surrounding 3D printing technology enables RamanBot to rapidly integrate hardware and software advancements, utilizing accessible control interfaces that offer superior interoperability compared to restrictive proprietary commercial software.

Future Kinematics: The current RamanBot design utilizes orthogonal incidence, a configuration suitable for the majority of planar and semi-planar samples. To address geometries requiring non-perpendicular excitation, future iterations will integrate multi-axis robotic kinematics similar to those emerging in non-planar 3D printing. For instance, [1] employed a high-precision six-axis robotic arm to facilitate six degrees of freedom (6-DoF), enabling complex translation and rotation for omnidirectional access. For this robot, however, they developed custom-made software to control the 6-DOF motion because such complexity doesn’t fit in the standard machine language G-code.

Section D: Lag time Vs Printer speed

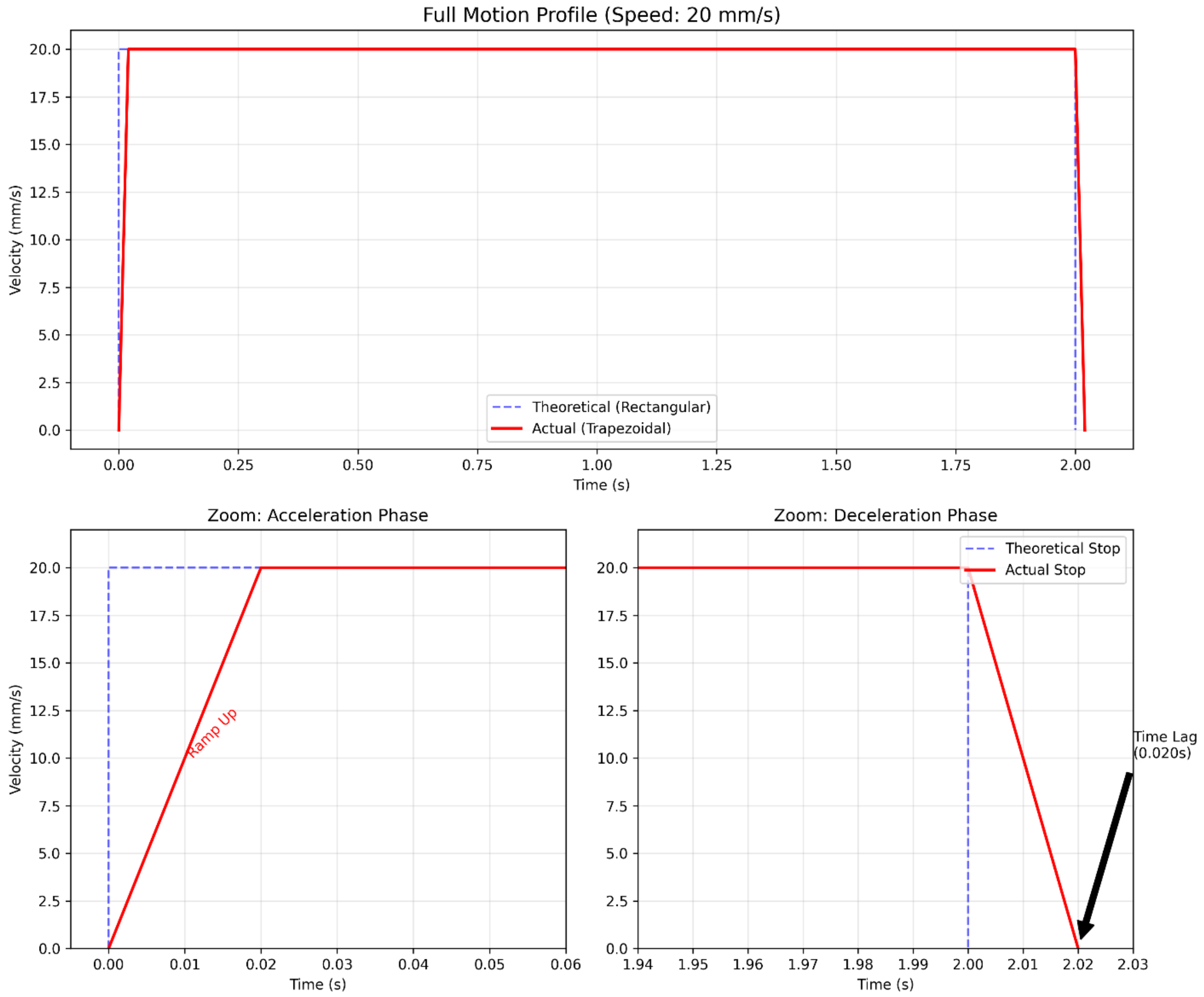

Figure D1: Comparison of theoretical (rectangular) and actual (trapezoidal) velocity profiles for a linear motion of 40 mm at a target velocity of 20 mm/s and acceleration of 1000 mm/s². (A) The complete velocity profile, showing minimal deviation between theoretical and actual total travel times. (B) Detailed view of the acceleration phase, illustrating the linear ramp-up required to reach target velocity. (C) Detailed view of the deceleration phase, highlighting the time lag ($\Delta t \approx 0.02\ s$) introduced by inertial constraints relative to the theoretical instant-stop model.

In our experiments, we used an additional margin time to accommodate for any differences between the actual travel time of the device and the theoretical travel time (commanded distance divided by the commanded speed). This difference arises

because the motion system cannot change velocity instantaneously due to mass and inertia. It must accelerate before reaching the targeted speed and decelerate before reaching a complete stop. Therefore, the actual travel time (t_{act}) can be expressed as

$$t_{act} = t_{acc} + t_{cru} + t_{dcc} \qquad \text{Eq. D1}$$

Where t_{acc} is the acceleration phase time, t_{cru} is the cruise phase time (constant speed), and t_{dcc} is the deceleration phase time. Figure D1 shows a comparison between the theoretical and actual model for a 3d printer. According to this equation, the difference between the actual travel time and the theoretical one is (v_c/a) where a is the acceleration and v_c is the commanded speed. Prusa i3 MK3S printer has a constant acceleration of 3000 mm/s^2. Therefore, the difference between the actual travel time and the theoretical travel time increases with the increase of the printing speed.

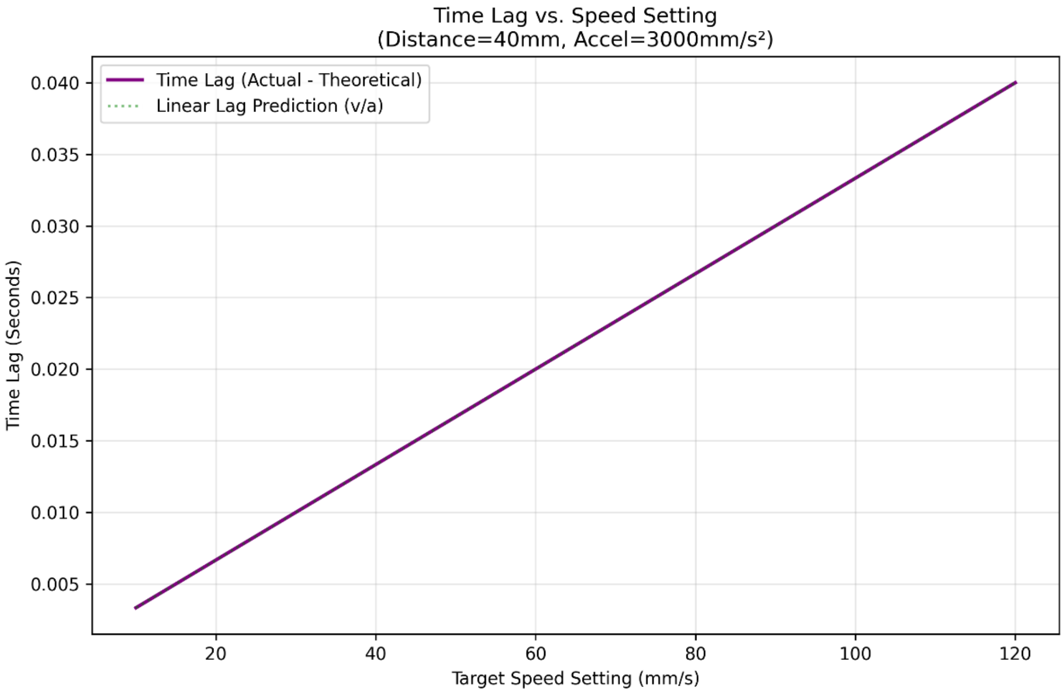

Figure D2: Temporal discrepancy in kinematic motion profiles. The plot illustrates the calculated time lag as a function of target velocity for a linear displacement of 40 mm with a fixed acceleration of 3000 mm/s².

Figure D2 shows the linear relation between the lag time and the speed. According to this theoretical study, the margin time should increase with the speed increase. The experimental values of this time lag, however, differ from the theoretical values due to frictions and imperfections in physical components of the system. Since we fixed the speed in our examples to 20 mm/s, we performed an experiment to compute the lag time using distance 50, 100, and 150 mm. We repeated the experiment for each distance 20 times from different random locations. The lag time was measured by analyzing the audio generated from the motors when it starts moving and when it stops.

	50 mm			100 mm			150 mm		
	mean	min	max	mean	min	max	mean	min	max
X lag	91 ms	55 ms	161 ms	93 ms	70 ms	128 ms	96 ms	49 ms	167 ms
Y lag	112 ms	91 ms	152 ms	115 ms	78 ms	135 ms	118 ms	94 ms	155 ms

Table D1: Statistical analysis of motion delay (Actual Time - Theoretical Time). Delays were measured for varying move lengths (50, 100, 150 mm) on both X and Y axes using audio signature analysis.

Table D1 shows the mean, min and max of the lag time in ms for each distance. We observed that the mean lag time increases slightly with the distance in X direction (91~96 ms) and the same for y (112~118 ms). Therefore, we used extended time margin (2 seconds) in centrifuge tube array and eggshells examples since the distance between samples are longer than for the microplate.

References

[1] J. Huang, H. O. T. Ware, R. Hai, G. Shao, and C. Sun, "Conformal Geometry and Multimaterial Additive Manufacturing through Freeform Transformation of Building Layers," *Adv. Mater.*, vol. 33, no. 11, Art. no. 2005672, Mar. 2021, doi: 10.1002/adma.202005672.
